# Physicochemical properties that control protein aggregation also determine whether a protein is retained or released from necrotic cells

**DOI:** 10.1098/rsob.160098

**Published:** 2016-11-03

**Authors:** Andre L. Samson, Bosco Ho, Amanda E. Au, Simone M. Schoenwaelder, Mark J. Smyth, Stephen P. Bottomley, Oded Kleifeld, Robert L. Medcalf

**Affiliations:** 1 Australian Centre for Blood Diseases, Alfred Medical Research and Education Precinct (AMREP), Monash University, Melbourne, Victoria 3004, Australia; 2 Department of Biochemistry and Molecular Biology, Monash University, Clayton, Victoria 3800, Australia; 3 Heart Research Institute, and Charles Perkins Centre, University of Sydney, Camperdown, New South Wales 2006, Australia; 4 Immunology in Cancer and Infection Laboratory, QIMR Berghofer Medical Research Institute, Herston, Queensland 4006, Australia; 5 School of Medicine, University of Queensland, Herston, Queensland 4006, Australia; 6 Faculty of Biology, Technion-Israel Institute of Technology, Haifa 3200003, Israel

**Keywords:** protein aggregation, necrosis, disulfide, immune tolerance, RNA-binding protein FUS, proteomics

## Abstract

Amyloidogenic protein aggregation impairs cell function and is a hallmark of many chronic degenerative disorders. Protein aggregation is also a major event during acute injury; however, unlike amyloidogenesis, the process of injury-induced protein aggregation remains largely undefined. To provide this insight, we profiled the insoluble proteome of several cell types after acute injury. These experiments show that the disulfide-driven process of nucleocytoplasmic coagulation (NCC) is the main form of injury-induced protein aggregation. NCC is mechanistically distinct from amyloidogenesis, but still broadly impairs cell function by promoting the aggregation of hundreds of abundant and essential intracellular proteins. A small proportion of the intracellular proteome resists NCC and is instead released from necrotic cells. Notably, the physicochemical properties of NCC-resistant proteins are contrary to those of NCC-sensitive proteins. These observations challenge the dogma that liberation of constituents during necrosis is anarchic. Rather, inherent physicochemical features including cysteine content, hydrophobicity and intrinsic disorder determine whether a protein is released from necrotic cells. Furthermore, as half of the identified NCC-resistant proteins are known autoantigens, we propose that physicochemical properties that control NCC also affect immune tolerance and other host responses important for the restoration of homeostasis after necrotic injury.

## 1. Introduction

Proteins must fold into their native conformation to be fully functional. However, as the native conformation is labile, all proteins are also capable of misfolding. Protein misfolding exposes hydrophobic regions that form intra- and inter-molecular associations, which if left unchecked can multimerize into higher-order toxic aggregates [1]. Protein quality control mechanisms adequately deal with most forms and degrees of misfolding. Nonetheless, destabilizing mutations, extreme environments or ageing can overwhelm proteostasis leading to the unwanted accumulation of aggregated proteins. Protein aggregation, and in particular amyloidogenic aggregation, underlies many chronic age-related diseases including Alzheimer's disease and Parkinson's disease. In these chronic settings, the onset of protein aggregation is widely thought to elicit injury.

Paradoxically, protein aggregation is also a key response to acute tissue injury. Injury-induced protein aggregation was first described in the 1980s [2–5], where aberrant actin aggregation occurred in cells after ATP depletion or oxidant exposure. Subsequent studies showed that actin, myosin, vinculin and HSP70 all aggregated upon ATP depletion [6–9]. Glyceraldehyde-3-phosphate dehydrogenase (GAPDH) is another intracellular protein that readily aggregates during stress *in vitro* [10–14] and *in vivo* [10,14–16]. Injury-induced protein aggregation has also been observed during hypoxia [17–19].

Recently, we [10,12,20,21] and others [22] have characterized the process of injury-induced protein aggregation. In particular, we found that injury can trigger a specific form of protein aggregation called nucleocytoplasmic coagulation (NCC) [10]. By definition, NCC is a temporally coordinated disulfide-crosslinking reaction that occurs during late-stage cell death. NCC causes many intracellular proteins, including actin, tubulin, GAPDH and HSP90β, to convert from a soluble form into high-molecular-weight insoluble species [10]. *In vitro* and *in vivo* data show that NCC-aggregated proteins promote activation of the extracellular protease plasmin on the surface of dead cells [10,20,21]. Surface-bound plasmin then initiates two forms of clearance: direct proteolytic degradation of dead cells [10,20] and signalling nearby dendritic cells to increase their phagocytic capacity [21]. Thus, NCC-aggregated proteins can be considered a novel ‘damage-associated molecular pattern’ that initiates humoral and cellular responses to sterile injury [23].

Despite these recent advancements the mechanisms of injury-induced protein aggregation are poorly understood. Accordingly, to ascertain the basis of injury-induced protein aggregation, we have used quantitative mass spectrometry to profile the insoluble proteome of three cell types across different injury conditions. We find that NCC is a specific consequence of necrosis. Moreover, our data suggest that NCC is the predominant form of injury-induced protein aggregation, which affects a much wider array of proteins than first thought. Surprisingly, a small subset of proteins avoids NCC-mediated aggregation. Comparison of NCC-resistant and NCC-sensitive proteins uncovers two fundamental principles: (i) that injury-induced aggregation is distinguishable from amyloidogenic aggregation, and (ii) that a protein's inherent physicochemical properties determines whether it is retained or released from necrotic cells.

Altogether, NCC represents a ‘fail-safe switch’ that broadly incapacitates cellular functions during necrosis and selectively retains debris at the site of injury. As a result, we propose that NCC is a beneficial event that pacifies the reactivity of necrotic cells. In support of this notion, and consistent with the fact that defective removal of dead cells is a strong risk factor for autoimmune disease [24–30], we note that many proteins which evade NCC are also recognized autoantigens. Future studies should now assess whether mutations that alter a protein's propensity to undergo NCC can in turn influence its ability to act as an autoantigen.

## 2. Results and discussion

### 2.1. Profiling the insoluble proteome of injured cells

**Figure 1.**
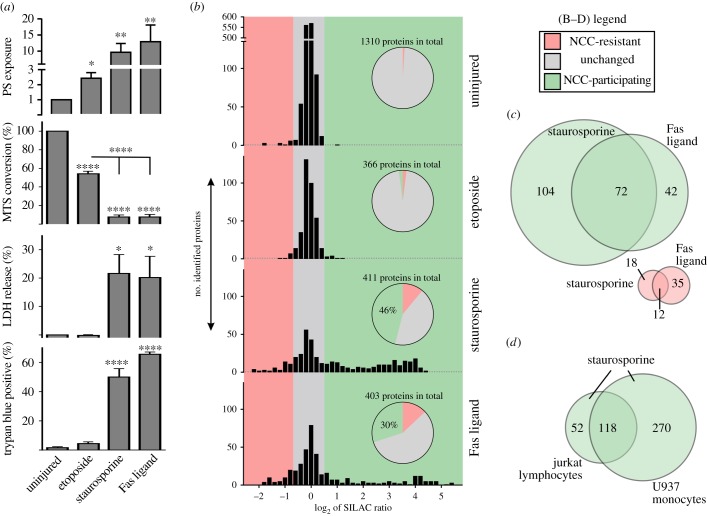
Diametric changes in the abundance of insoluble intracellular proteins upon secondary necrosis. (*a*) Jurkat lymphocytes were treated with vehicle (uninjured) or injured with etoposide, staurosporine or Fas ligand. Phosphatidylserine (PS) exposure (relative AnnexinV-signal geomean), metabolism (% MTS) and necrosis (% LDH and % trypan blue positive) were measured after 24 h of treatment. Data are presented as mean + s.e.m. from *n* = 3–7 independent experiments. *p < 0.05, **p < 0.01 and ****p < 0.0001 relative to uninjured cultures as determined by one-way ANOVA with Newman–Keuls correction. By definition, apoptosis is an energetic process, whereas necrosis coincides with metabolic incompetency. Therefore, because 24 h incubation with staurosporine or Fas ligand abolished metabolism (in conjunction with high levels of LDH release, trypan blue uptake and TO-PRO-3-staining; electronic supplementary material, figure S1*a*), we have concluded that these agonists produced a secondary necrotic state by this time point. (*b*) Jurkat cultures were labelled with either heavy or light lysine isotopes and treated with vehicle (uninjured), etoposide, staurosporine or Fas ligand for 24 h. Triton-insoluble fractions were extracted and proteins identified using quantitative mass spectrometry. The abundance of identified proteins relative to that in isotopically different uninjured cultures was then plotted as the log_2_ ratio of the stable isotope label (SILAC); where an increased ratio indicates more of a specific insoluble protein in injured cells relative to uninjured cells and a decreased ratio indicates less of a specific insoluble protein in injured cells relative to uninjured cells. Proteins with a log_2_ ratio > +0.6 were gated into the NCC-participating subproteome (green colour) and proteins with a log_2_ ratio < −0.6 were gated into the NCC-resistant subproteome (pink colour). This gating was also chosen based on the recommendations of Zhang & Neubert [31]. Inserts show the percentage of identified proteins that fall within each gate. (*c*) Partial overlap in the gated subproteomes between staurosporine- and Fas ligand-induced necrotic Jurkat cells. The number of proteins within each gate is shown. (*d*) Partial overlap in the NCC-participating subproteome between staurosporine-induced necrotic Jurkat cells versus staurosporine-induced necrotic human U937 monocytes.

We first characterized injury and protein aggregation in human Jurkat lymphocyte cells treated with etoposide (a topoisomerase-II inhibitor), staurosporine (a pan-kinase inhibitor) or Fas ligand (FasL; a TNF death receptor agonist). All agonists induced apoptosis in Jurkat cells (indicated by an increase in phosphatidylserine exposure and caspase-mediated RIPK1 cleavage; figure 1*a*; electronic supplementary material, figure S1*a*,*b*). More specifically, FasL and staurosporine triggered a similar degree/rate of injury, with early stage apoptosis evident within 3 h of treatment (indicated by phosphatidylserine-positive, TO-PRO-3-negative staining; electronic supplementary material, figure S1*a*) and transition to a secondary necrotic state within 24 h of treatment (indicated by complete loss of metabolism, plasma membrane disruption, pronounced LDH release and the acquirement of TO-PRO-3-positive staining; figure 1*a*; electronic supplementary material, figure S1*a*). Note that neither necroptosis (mediated by full-length RIPK1) nor autophagic death (mediated by LC3 cleavage; electronic supplementary material, figure S1*b*) was evident after FasL or staurosporine treatment. By contrast, etoposide caused early stage apoptosis after 24 h of treatment with no signs of secondary necrosis (as indicated by LDH retention, plasma membrane impermeability, partial metabolic suppression, and modest elevations in phosphatidylserine exposure and RIPK1 cleavage; figure 1*a*; electronic supplementary material, figure S1*a*). Lastly, we confirmed protein aggregation in Jurkat cells during the later stages of staurosporine-treatment with synchronous conversion of HSP90β, α/β-tubulin and β-actin from a soluble state into insoluble high molecular weight disulfide-crosslinked species (electronic supplementary material, figure S1*c*). Disulfide-crosslinked insoluble forms of these same proteins were also observed after FasL treatment, but not after etoposide treatment (not shown). Note that the coordinated loss of protein solubility upon secondary necrosis represents a genuine aggregation event (rather than merely being a consequence of disulfide crosslinking), as these same insoluble proteins were observed in the presence of a reducing agent (electronic supplementary material, figure S1*d*). Thus, injury-induced protein aggregation in Jurkat cells occurred specifically during necrosis, but not during early stage apoptosis. Moreover, as the vast majority of insoluble material was disulfide-crosslinked (electronic supplementary material, figure S1*c*), NCC appears to be the predominant form of injury-induced protein aggregation.

**Figure 2.**
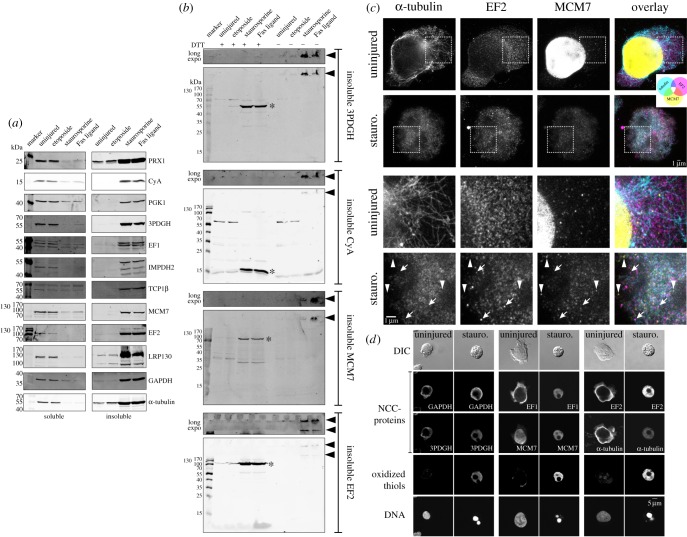
NCC is the main form of injury-induced protein aggregation. (*a*–*d*) Jurkat cells were treated with vehicle (uninjured), etoposide, staurosporine or Fas ligand for 24 h. (*a*) Triton-soluble and -insoluble proteins were isolated and subjected to SDS-PAGE under reducing conditions followed by immunoblot analysis. Results are representative of *n* = 3–6 independent experiments. (*b*) Triton-soluble and -insoluble proteins were isolated and subjected to SDS-PAGE in the presence/absence of dithiothreitol (DTT) followed by immunoblot analysis. Results are representative of *n* = 2–3 independent experiments. (*c*) Cells were fixed, subjected to immunofluorescence and imaged by super-resolution microscopy with *xy-*resolution of 70 nm. Shown are representative *x*,*y* micrographs, where the bottom two rows are magnified micrographs taken from the boxed areas in the top two rows. Staurosporine-induced necrosis causes NCC-proteins (α-tubulin, EF2 and MCM7) to relocate into aberrant and distinct structures. Arrows indicate EF2 deposits that exhibit little-to-no staining for tubulin or MCM7. Arrowheads indicate MCM7 deposits that exhibit little-to-no staining for tubulin or EF2. Note, the procedure used to stain oxidized thiols was not compatible with the sample preparation for super-resolution microscopy. (*d*) Cells were fixed and subjected to immunofluorescence with maleimide co-staining for oxidized thiols. Shown are representative *x,y* confocal micrographs. Staurosporine-induced necrosis leads to nuclear breakdown and a marked increase in oxidized thiols that colocalize with aberrantly deposited NCC-proteins (3PDGH, GAPDH, MCM7, EF1, EF2 and α-tubulin).

Next, we profiled the insoluble proteome of Jurkat cells after 24 h of treatment with vehicle, etoposide, staurosporine or FasL using the quantitative mass spectrometry-based technique of SILAC (stable isotope labelling by amino acids in cell culture [32]; see electronic supplementary material, figure S2 and Material and methods for experimental details). This approach allowed the identification of hundreds to thousands of insoluble proteins per experiment with measurement of their relative abundance between uninjured and injured cells. Figure 1*b* and electronic supplementary material, table S1 summarize the SILAC results. Proteins with an increased right-shifted SILAC ratio in figure 1*b* were assumed to have undergone injury-induced aggregation, as this shift was only observed in staurosporine- or FasL-treated Jurkat cells. To verify this assumption, 12 proteins identified as putative aggregators in the SILAC experiments were subjected to immunoblot analysis. As shown in figure 2*a*, all 12 proteins were confirmed to undergo injury-induced protein aggregation in necrotic Jurkat cells. Consistent with the initial description of NCC in neuronal cells [10], necrosis in Jurkat cells caused all tested proteins to convert into insoluble high-molecular-weight dithiothreitol-sensitive species (as assessed by immunoblot; figure 2*b*) and to relocate into distinct aberrant subcellular structures (as assessed by super-resolution microscopy; figure 2*c*) which stained strongly for the presence of oxidized thiols (as assessed by confocal microscopy; figure 2*d*). Thus, we concluded that proteins that exhibited log_2_ SILAC ratios greater than +0.6 were indeed ‘NCC-participating’ proteins (green colour in figure 1*b*). Moreover, as 30–46% of the identified proteins underwent NCC in necrotic Jurkat cells (figure 1*b*), injury-induced aggregation appears to affect a much larger portion of the proteome than prior studies suggest.

**Figure 3.**
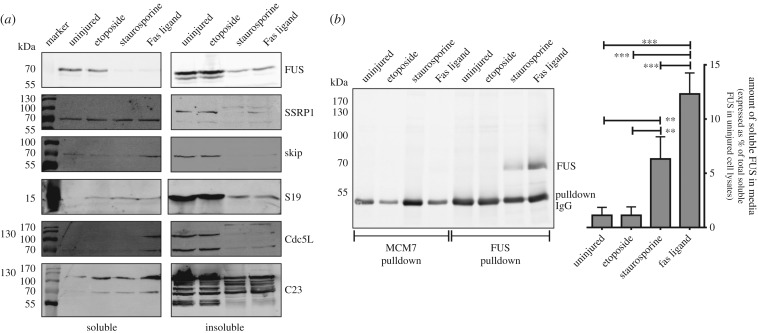
A subset of intracellular proteins avoids NCC and is released into the extracellular milieu upon necrosis. (*a*,*b*) Jurkat cells were treated with vehicle (uninjured), etoposide, staurosporine or Fas ligand for 24 h. (*a*) Triton-soluble and -insoluble proteins were isolated and subjected to SDS-PAGE under reducing conditions followed by immunoblot analysis. Results are representative of *n* = 3–5 independent experiments. (*b*) Conditioned media were collected and the amount of MCM7 (an NCC-participating protein) and FUS (an NCC-resistant protein) assayed via immunoprecipitation and subsequent immunoblotting (simultaneously using primary antibodies for both MCM7 and FUS). The approximately 50 kDa immunoreactive species represents the long-chain immunoglobulin used for the immunoprecipitation. The approximately 70 kDa immunoreactive species represents full-length FUS. No MCM7 (an approx. 100 kDa protein) was detected in the conditioned media. The graph depicts the collated quantitation of the immunoprecipitated FUS signal normalized to the total amount of soluble full-length FUS in uninjured cells. Data are presented as mean + s.e.m. from *n* = 4 independent experiments. **p < 0.01 and ***p < 0.001 as determined by one-way ANOVA with Newman–Keuls correction.

**Figure 4.**
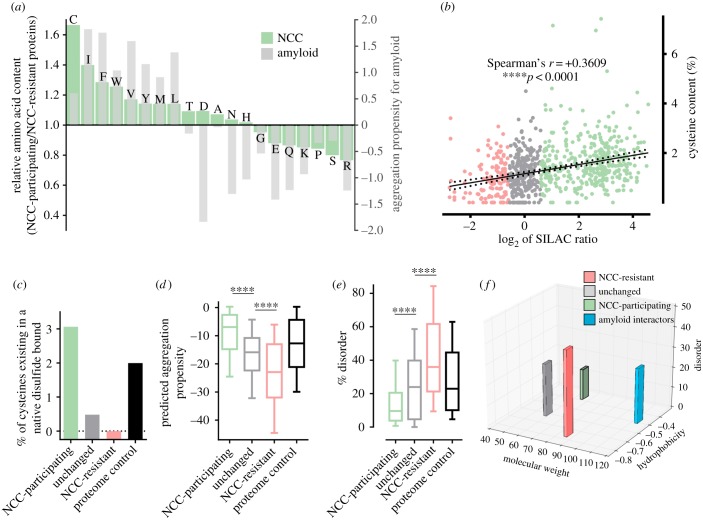
NCC has defining physicochemical properties that distinguish it from amyloidogenic aggregation. (*a*–*f*) A high-confidence list of 388 NCC-participating, 128 NCC-resistant and 262 intermediary ‘unchanged’ proteins was collated from the Jurkat and U937 SILAC datasets (electronic supplementary material, table S1). Only proteins quantified in at least two necrotic instances were considered. A fourth subset of 388 proteins randomly selected from the reviewed human nuclear/cytoplasmic/mitochondrial proteome was also generated for control purposes (electronic supplementary material, table S1). The four subsets were used for physico-chemical trait analysis. (*a*) Shown in green is the amino acid composition of NCC-participating proteins expressed relative to that of NCC-resistant proteins (arbitrarily normalized to 1). Shown in grey is the predicted relative contribution of individual amino acids to amyloidogenic aggregation as defined in AGGRESCAN [33] (arbitrarily normalized to 0). (*b*) Graph depicting a significant (*p* < 0.0001) positive correlation between the relative cysteine content and the tendency for proteins to undergo NCC (i.e. SILAC ratio) as determined by Pearson's correlation analysis (where the black solid line represents the line of best fit and black dashed lines indicate 95% confidence intervals). (*c*–*e*) Graphs comparing the degree of native disulfide bonding (*c*), the predicted aggregation propensity as determined by AGGRESCAN [33] (*d*) and the intrinsic disorder content (*e*) across the four protein subsets. For the box plots in (*d*,*e*), the centre line indicates the median, box edges indicate the 25th/75th percentile and whiskers indicate the 10th/90th percentile. ****p < 0.0001 as determined by one-way ANOVA with Tukey's correction. (*f*) Graph comparing the average molecular weight (kDa), hydrophobicity and % disorder for the NCC-participating, NCC-resistant and unchanged protein subsets with that of amyloid interacting proteins identified in [34] (another protein subset from electronic supplementary material, table S1).

**Figure 5.**
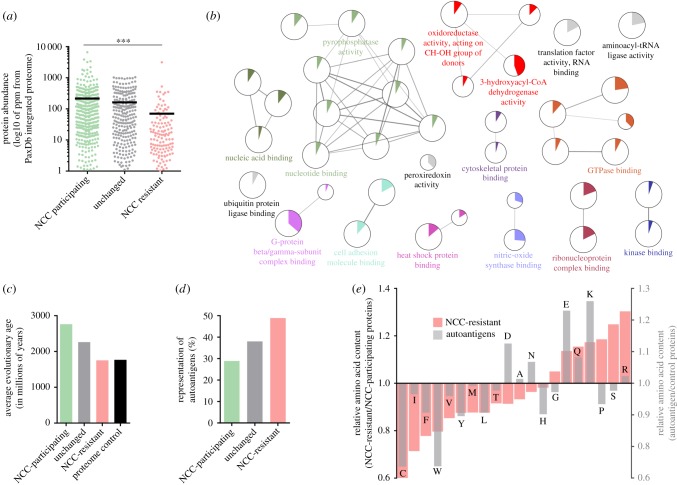
NCC preferentially affects abundant ancient proteins that perform essential cellular functions and exhibit reduced autoreactivity. (*a*–*e*) The same high-confidence protein subsets and the randomly selected control subset from figure 4 were further analysed. (*a*) Graph comparing the abundance across the different protein subsets. Black lines indicate the average abundance of each subset. ***p < 0.001 as determined by one-way ANOVA with Tukey's correction. (*b*) Network depicting the molecular functions that were significantly over-represented in the NCC-participating subset, relative to the complete human proteome. Nodes (circles) represent different molecular function ontologies. Functionally related nodes are connected by edges (straight lines) and have been grouped using ClueGO [35], *κ* ≥ 0.4. The node size is proportional to the statistical significance of enrichment. The coloured pie segments that fill each node indicate the per cent of the ontology that appears in the NCC-participating subset. The complete list of enriched molecular functions (with statistical significance values) for both the NCC-participating and NCC-resistant subsets is provided in electronic supplementary material, table S2. (*c*,*d*) Graphs comparing the average evolutionary age and the representation of known autoantigens within the different protein subsets. (*e*) Shown in pink is the amino acid composition of NCC-resisting proteins expressed relative to that of NCC-participating proteins (arbitrarily normalized to 1). Shown in grey is the amino acid composition of known autoantigens (expressed relative to amino acid composition of a set of control proteins provided by Backes *et al*. [36]).

Closer inspection of the SILAC data revealed another population of proteins with log_2_ SILAC ratios less than −0.6 (11–13% of identified targets; red colour in figure 1*b*). These ‘NCC-resistant’ proteins were decreased in abundance in the insoluble fraction of necrotic Jurkat cells relative to uninjured cells, suggesting that they were either degraded or avoided aggregation during injury. To assess which scenario was applicable, six putative NCC-resistant proteins were selected for immunoblot analysis. Consistent with the SILAC data, all six tested proteins were decreased in abundance in the insoluble fraction of necrotic Jurkat cells relative to uninjured cells (figure 3*a*). Notably, the disappearance of these NCC-resistant proteins in necrotic cells did not result in the commensurate appearance of lower molecular weight immunoreactive species, suggesting that they were not being degraded (figure 3*a*). Next, we reasoned that if a protein avoided both NCC and degradation, then it should instead be released into the extracellular milieu upon necrosis. Consequently, we assayed for the presence of FUS (also called ‘RNA-binding protein FUS’ or ‘Translocated in liposarcoma’, TLS; which was the most easily detected NCC-resistant protein) in the conditioned media from injured Jurkat cells. As shown in figure 3*b*, full-length FUS, but not MCM7 (an NCC-participating protein), was readily detected in the conditioned media of necrotic Jurkat cells. To the best of our knowledge, this is the first report that the ubiquitous protein, FUS, is released from necrotic cells. It remains to be determined whether other NCC-resistant proteins are released from necrotic cells, but this seems highly likely (for reasons provided below with figures 4 and 5). The capacity of FUS and other NCC-resistant proteins to act as paracrine communicators of injury should now be assessed, especially given the influence of NCC on the proteolytic [10,20] and phagocytic [21] removal of dead cells. It will also be interesting to see if FUS released from necrotic cells has a role during FUS-mediated amyotrophic lateral sclerosis [37].

We next examined injury-induced protein aggregation in human U937 monocyte cells, where both secondary necrosis (electronic supplementary material, figure S3*a*) and NCC (electronic supplementary material, figure S3*b*) occurred after 24 h of treatment with either etoposide or staurosporine. SILAC experiments showed that similar, albeit more pronounced changes to the insoluble proteome occurred upon secondary necrosis in U937 cells (figure 1*d*; electronic supplementary material, figure S3*c*), with 53–61% of proteins participating in NCC and 16–30% of proteins avoiding NCC (electronic supplementary material, figure S3*c*). The reason for this increased aggregation of the U937 proteome is unknown, but may reflect the heightened sensitivity of U937 cells to injury (cf. figure 1*a* and electronic supplementary material, figure S3*a*), or could relate to the extreme differences in the metabolism of monocytes and lymphocytes [38]. It is telling that etoposide elicited neither necrosis nor NCC in Jurkat cells, but strongly induced both secondary necrosis and NCC in U937 cells. This observation emphasizes that necrosis *per se* is the major cellular correlate of NCC. It is also salient that, despite marked differences in the means by which etoposide, staurosporine and FasL injure cells, many of the same proteins underwent NCC when these treatments caused necrosis (figure 1*c*; electronic supplementary material, figure S3*d*). Such significant overlap in the insoluble proteome across different cell types and necrotic conditions denotes that a common underlying mechanism governs the participation/resistance of proteins to NCC.

### 2.2. Elucidating the physicochemical basis of nucleocytoplasmic coagulation

All proteins are capable of aggregating; however, the propensity, kinetics and conformations of aggregation are largely determined by the inherent physicochemical properties of the protein in question. Therefore, a high-confidence list of NCC-participating, NCC-resistant and intermediary ‘unchanged’ proteins was collated from the Jurkat and U937 SILAC datasets (electronic supplementary material, table S1), and their primary sequences were compared to ascertain the physicochemical basis of NCC. The most prominent feature of NCC-participating proteins was their relatively high cysteine content (figure 4*a*), with a significant correlation between cysteine content and the degree of injury-induced aggregation (figure 4*b*). This finding, in conjunction with our earlier publications showing that NCC-aggregates are disulfide-crosslinked [10,12], highlights the importance of cysteine residues to NCC. Conversely, NCC-resistant proteins were found to be devoid of native disulfide bonds (figure 4*c*), implying that unlike NCC-participating proteins, the functions/folding of NCC-resistant proteins may be independent of cysteine reactivity. With the exception of C, D, N and H residues, the amino acid content of NCC-participating proteins aligned well with that of amyloidogenic peptides, as defined in [33,39,40] (figure 4*a* and not shown). This observation suggests that amyloidogenic aggregation and NCC are similar, but nevertheless distinct processes. We propose that NCC is an ‘off-shoot’ of amyloidogenic aggregation (electronic supplementary material, figure S4). NCC and amyloidogenesis may even be at cross-purposes, given that amyloidogenesis involves the slow organized association of discrete protein segments in the absence of disulfide crosslinking, whereas NCC involves rapid multivalent associations in the presence of disulfide crosslinking (electronic supplementary material, figure S4). Further evidence for this hypothesis stems from our prior work showing that NCC-aggregates exhibit a prefibrillar oligomeric conformation with no subsequent appearance of fibrillar species [10].

Other characteristics of NCC-participating proteins include a higher representation of aromatic residues (F, Y, W; *p* < 0.001 via unpaired two-tailed *t*-test; figure 4*a*), aliphatic residues (I, V, L; *p* < 0.0001 via unpaired 2-tailed *t*-test; figure 4*a*) and increased hydrophobicity (electronic supplementary material, figure S5*a*)—attributes which explain the predicted high aggregation propensity of NCC-participating proteins relative to NCC-resistant proteins (figure 4*d*). Curiously, the intrinsic disorder content (figure 4*e*) and the molecular weight (electronic supplementary material, figure S5*b*) of NCC-participating proteins were lower than those of NCC-resistant proteins. These collective attributes further distinguish NCC from amyloidogenesis, which instead favours co-aggregation of large hydrophobic proteins with high intrinsic disorder (figure 4*f*) [34]. That diametrically opposed traits exist between NCC-participating and NCC-resistant proteins shows that the same physicochemical forces control these protein fates during necrosis. Indeed, our data show that whether a protein is retained or released from necrotic cells is in part determined by its propensity to undergo injury-induced aggregation. This conclusion was independently supported by analysing the insoluble proteome of a third cell line after necrotic injury (i.e. the mouse AT3 mammary adenocarcinoma cells; electronic supplementary material, figure S6 and table S1).

### 2.3. Identifying higher-order features of nucleocytoplasmic coagulation

Many factors besides primary sequence affect protein aggregation. Foremost among these factors is the concentration dependency of protein aggregation. In line with this tenet, NCC-participating proteins were found to be more abundant than NCC-resistant proteins (figure 5*a*). Next, we performed ontology enrichment analysis to gain a broader sense of what molecular functions (figure 5*b*; electronic supplementary material, table S2) and subcellular compartments (electronic supplementary material, figure S7*a* and table S3) were affected by NCC. These analyses showed that NCC preferentially affects metabolic (e.g. GTPases), oxidoreductase (e.g. dehydrogenases, periorodoxins), signalling (e.g. kinases) and proteostatic enzymes (e.g. HSPs, translation factors), as well as nucleotide- and nucleic acid-binding proteins (*p* < 0.05; electronic supplementary material, table S2). NCC was also found to preferentially affect macromolecular complexes in the nucleus (e.g. nuclear pore), cytoplasm (e.g. chaperonin, proteosome, cytoskeleton) and mitochondria (electronic supplementary material, figure S7*a* and table S3), subcellular compartments with a constitutively redox-reduced state (*p* < 0.05; electronic supplementary material, table S3). By comparison, distinct molecular functions and subcellular compartments were prevalent among NCC-resistant proteins (electronic supplementary material, tables S2 and S3). Thus, numerous energetic and homeostatic functions in the nucleus, cytoplasm and mitochondria are directly affected by NCC.

Interrogation of the human interactome [41] suggests that NCC causes aggregation of seemingly unrelated proteins (i.e. the NCC-interactome has low connectivity; electronic supplementary material, figure S7*b*) that participate in a high number of protein–protein interactions (especially, homotypic interactions; electronic supplementary material, figure S7*b*). That NCC preferentially affects fundamental enzyme complexes agrees well with the observation that NCC-participating proteins are evolutionarily more ancient than NCC-resistant proteins (figure 5*c*). Altogether, our data suggest that NCC preferentially shuts down a wide array of ancient proteins with essential interactions and functions. NCC is therefore likely to be a rudimentary response that ‘inactivates’ necrotic cells.

### 2.4. Putative role for nucleocytoplasmic coagulation in attenuating autoimmunity

Maintaining tolerance after injury is a necessary and problematic process, especially given that cell death can allow the unwanted exposure/release of epitopes that would otherwise be sequestered from the immune system. Indeed, cell death is a potent trigger of inflammation, with autoimmune disease often preceded by injury [24–27]. Moreover, defective or inadequate removal of dead cells underlies the onset of many autoimmune disorders including system lupus erythematosus [28]. Accordingly, we hypothesized that NCC, by restricting the escape of constituents from necrotic cells and by promoting tolerogenic clearance [21], is a favourable response that mitigates autoimmunity. To support this hypothesis, we assessed the representation of known autoantigens (using a set of 2079 autoantigens from Backes *et al*. [36]) and found that autoantigens were far more prevalent among the NCC-resistant subpopulation (figure 5*d*). Furthermore, the primary amino acid composition of autoantigens closely mirrored that of NCC-resistant proteins, with cysteines (*p* < 0.0001), aliphatic residues (I, V, L; *p* < 0.0001) and aromatic residues (F, Y, W; *p* < 0.0001) being under-represented, and charged residues (D, E, K, R; *p* < 0.0001) being over-represented in autoantigens (figure 5*e*). Collectively, this profile may allow autoantigens to evade NCC and thereby promote their release into the extracellular space upon necrosis. Collectively, these findings add support to the hypothesis that NCC is a favourable response that may mitigate autoimmunity. Future studies should now test whether mutations that increase a protein's tendency to undergo NCC also reduce its autoreactivity following necrosis—an approach that holds great appeal for investigating the basis of lupus and other injury-related autoimmune disorders.

### 2.5. Concluding remarks

A cascade of events that accounts for both our previous [10,12] and current findings is where NCC relies upon membrane disruption during necrosis (either primary or secondary necrosis). Membrane failure would cause a major loss of redox potential and widespread oxidation, which in turn would preferentially disulfide-crosslink abundant protein complexes within otherwise redox-reduced compartments. Our prior studies also suggest that initial misfolding events (e.g. linchpin residue oxidation) that precede necrosis may be a prerequisite for NCC [10,12]. We postulate that NCC represents a fail-safe switch that ensures irreversible metabolic and proteostatic incompetency during intense injury. The proteomic coverage of NCC is much wider than first thought, with a large number of essential proteins and macromolecular structures entrapped by NCC, thereby preventing their release into the extracellular space. Entrapment by NCC, however, is a selective process because a small proportion of the proteome (including FUS) avoids NCC and is therefore selectively released into the extracellular milieu upon necrosis. As the release of intracellular molecules upon necrosis is deleterious, NCC is likely to be a beneficial response that helps multicellular organisms restore tissue homeostasis after injury. This supposition is somewhat counterintuitive given that amyloidogenic protein aggregation promotes neurodegenerative diseases. However, based on the similar albeit distinct physicochemical signatures of NCC and amyloidogenesis, we hypothesize that NCC may be an ‘offshoot pathway’ that prevents deleterious amyloid formation in necrotic cells (electronic supplementary material, figure S4). Such interrelations between amyloidogenic aggregation and NCC should now be assessed. Beyond this, it is intriguing that features such as cysteine content, hydrophobicity, intrinsic disorder and abundance dictate whether a protein is retained or released from necrotic cells. This understanding broadens the biology of protein aggregation and indicates that reductionist biochemical approaches used in the field of protein misfolding may also be used to investigate the immunogenic and inflammatory nature of necrosis.

## 3. Material and methods

### 3.1. Materials

Reagents were from Sigma unless otherwise indicated. Soluble Fas ligand (*Super*FasLigand) was from Enzo Life Science. NuPAGE Novex Bis-Tris Mini gel (4–12%), NuPAGE MOPS SDS Running buffer, RPMI media, penicillin and streptomycin, dialysed and non-dialysed fetal calf serum (FCS), TO-PRO-3 and 7-AAD were from Life Technologies. Immobilon-FL polyvinylidene difluoride (PVDF) membrane and Protein G Plus/Protein A agarose (50% suspension) beads were from Merck Millipore. Cell culture SILAC reagents were from Cambridge Isotope Laboratories. InstantBlue protein stain was from CBS Scientific. Sequence Grade Modified Trypsin (34 V511A), CytoTox96 non-radioactive cytotoxicity (LDH) and CellTitre96 aqueous non-radioactive cell proliferation (MTS) assays were from Promega. Odyssey Blocking Buffer and IRdye secondary antibodies (800CW donkey anti-mouse IgG, 680LT donkey anti-mouse IgG, 800CW donkey anti-rabbit IgG, 680LT donkey anti-rabbit IgG, 800CW donkey anti-goat IgG and 680LT donkey anti-goat IgG) were from LI-COR Biosciences. Primary antibodies were from Santa Cruz Biotechnology unless stipulated otherwise: anti-RIPK1 (BD Biosciences, #610458), anti-LC3 (MBT International, #PM036), mouse anti-HSP90β (Abcam, #ab82522), rabbit anti-α-tubulin (Merck Millipore, #04–1117), mouse anti-β-tubulin (Sigma,#T0198), goat anti-β-actin (#sc-1616), goat anti-PRX1 (#sc-7381), rabbit anti-Cyclophilin A (Abcam, #ab41684), goat anti-PGK1 (#sc-17943), mouse anti-3PDGH (#sc-100317), rabbit anti-EF1 (#sc-28578), goat anti-IMPDH2 (Abnova, #PAB19702), mouse anti-TCP1β (#sc-373769), mouse anti-MCM7 (#sc-56324), goat anti-EF2 (#sc-13004), mouse anti-LRP130 (#sc-166178), mouse anti-GAPDH (Merck Millipore, #MAB374), mouse anti-FUS/TLS (#sc-373698), mouse anti-SSRP1 (#sc-56781), mouse anti-Skip (#sc-136546), rabbit anti-ribosomal protein S19 (#sc-134779), mouse anti-Cdc5 L (#sc-81220), mouse anti-C23 (#sc-8031) and rabbit anti-GAPDH (#sc-25778).

### 3.2. Cell culture

Jurkat and U937 cells were maintained as non-adherent cultures in RPMI supplemented with 10%(v/v) heat-inactivated FCS, 2 mM l-glutamine, 50 U ml^−1^ penicillin and 50 U ml^−1^ streptomycin (P/S) under humidified 5% CO_2_. AT3 cells were maintained as an adherent culture in DMEM supplemented with 10%(v/v) heat-inactivated FCS, 2 mM l-glutamine, 50 U ml^−1^ penicillin and 50 U ml^−1^ streptomycin (P/S) under humidified 5% CO_2_. For injury induction, cells were resuspend cells in RPMI/DMEM supplemented with 1%(v/v) heat-inactivated FCS, P/S and l-glutamine. Cells were then seeded at 0.15 × 10^6^ live cells per well (for Jurkat and AT3 cells) or 0.1 × 10^6^ live cells per well (for U937 cells) in 24-well plates and allowed to equilibrate for 1 h. Injury-causing treatments or an equivalent amount of DMSO (as the vehicle) were then added to their final concentrations: 300 nM staurosporine, 25 µg ml^−1^ etoposide (for Jurkat cells) or 6 µg ml^−1^ etoposide (for U937 cells), 40 ng ml^−1^ soluble Fas ligand and 500 nM doxorubicin. Injury was assessed 3–24 h later (as stipulated in the corresponding figure legend). For stable Lysine isotope incorporation, cells were maintained for more than 10 passages in Lysine- and Arginine-free RPMI/DMEM media supplemented with 10% dialysed FCS, P/S, l-glutamine, 0.1 g l^−1^ of unlabelled l-Arginine : HCl and either 0.1 g l^−1^ of unlabelled l-Lysine : 2HCl or 0.1 g l^−1^ of l-Lysine : 2HCl (U-^13^C_6_, 99%).

### 3.3. Cell injury assays (LDH, MTS and trypan blue assays)

LDH and MTS assay kits were from Promega and were performed according to the manufacturer's instructions. Trypan blue positivity was determined using the TC-10 Automated Cell counter (Bio-Rad) according to the manufacturer's instructions.

### 3.4. Flow cytometry

Jurkat and U937 cells (seeded and treated as in §3.2) were incubated for the indicated time period. Cells were then incubated for a further 5 min with 1 mg l^−1^ AlexaFluor488 conjugated-Annexin V and 250 µM TO-PRO-3 or 7-AAD and subject to flow cytometry (BD Accuri C6 Plus flow cytometer). Data were analysed off-line using FlowJo v. 10.1r5.

### 3.5. Cell homogenization and fractionation

Cells were collected, washed in PBS, pelleted and resuspended in ice-cold lysis buffer (PBS + 1% Triton X-100 + 1× complete EDTA-free protease cocktail (Roche) + 1× PhosSTOP phosphatase inhibitor cocktail (Roche) + 10 mM chloroacetamide). Chloroacetamide was added to the lysis buffer to prevent inadvertent disulfide bond formation during/after cell homogenization and subsequent fractionation. Note, chloroacetamide was chosen over iodoacetamide as it reacts more specifically with cysteine thiols [42]. Homogenates were triturated and then stored at −80°C until further fractionation was necessary; 1 ml of lysis buffer was used per 0.6 × 10^6^ cells (for Jurkat cells) or per 0.4 × 10^6^ cells (for U937 cells). To extract the soluble and insoluble fractions, 1 ml of homogenate was centrifuged (4°C, 16 000*g*, 30 min). The supernatant was kept as the ‘Triton-soluble fraction’ and the pellet was washed in 1 ml of PBS + 1% Triton X-100 and incubated for 1 h with gentle mixing at 25°C in 60 µl of 62.5 mM Tris–HCl (pH 6.8), 2% SDS, 5% glycerol with or without 0.1 M DTT. Finally, the sample was centrifuged (25°C, 16 000*g*, 30 min) and the supernatant kept as the ‘Triton-insoluble fraction’.

### 3.6. SDS-PAGE

#### 3.6.1. SDS-PAGE for immunoblotting

Samples (50 µl of Triton-soluble fraction and the entire Triton-insoluble fraction) were boiled in 2% SDS-loading buffer with or without dithiothreitol, subjected to SDS-PAGE, and transferred onto PVDF membranes. Membranes were probed with the appropriate IR-dye-conjugated secondary antibody (LI-COR) and signals revealed with an Odyssey scanner (LI-COR).

#### 3.6.2. SDS-PAGE for tryptic digestion

Samples were boiled in 2% SDS-loading buffer with dithiothreitol and subjected to SDS-PAGE using a NuPAGE Novex Bis-Tris Mini gel (4–12%) with NuPAGE MOPS SDS-Running buffer supplemented with 1× NuPAGE antioxidant (Life Technologies). After electrophoresis, the gel was stained with InstantBlue protein stain and cut into approximately 2 × 6 mm slices then each slice further cut into 1 mm^2^ pieces. Gel pieces were subjected to in-gel trypsin digestion as in [43].

### 3.7. Mass spectrometry

Liquid chromatography tandem mass spectrometry was performed as in [44] with minor modifications. Extracted peptides were analysed by Q-Exactive Orbitrap mass spectrometer (Thermo Scientific) coupled online with an RSLC nano-HPLC (Ultimate 3000, Thermo Scientific). Samples were injected onto a Thermo RSLC pepmap100, 75um id, 100 angstrom pore size, 50 cm reversed phase nano column with buffer A (2.5% acetonitrile, 0.1% formic acid) at a flow rate of 300 nl min^−1^. The peptides were eluted over a 40-min gradient to 40% buffer B (80% acetonitrile 0.1%, formic acid). The eluate was nebulized and ionized using the Thermo nano electrospray source coated silica emitter with a capillary voltage of 1700 V. Peptides were selected for MS/MS analysis using Xcalibur software (Thermo Fisher) in Full MS/dd-MS2 (TopN) mode with the following parameter settings: TopN 10, MSMS AGC target 1e5, 60 ms Max IT, NCE 27 and 2 *m*/*z* isolation window. Dynamic exclusion was set to 20 s.

MS data were analysed with MaxQuant [45] software v. 1.3.0.5. Search parameters included: specific digestion with trypsin with up to two missed cleavages, +6 Lysine was set as label, protein N-terminal acetylation and methionine oxidation were set as variable modifications while cysteine alkylation was set as fixed modification. The searches were performed against UniProt human protein sequences (March 2013 version). The remaining search parameters were default settings. The search was then imported into Perseus software [45] for collation (electronic supplementary material, table S1) and for further annotation. Note that the gating of SILAC ratios for analysis is explained in the respective figure legends. These gating strategies were based on the recommendations of Zhang & Neubert [31], with more than 95% of the identified proteins having a ratio that fell within the ‘unchanged’ intermediary gate in the corresponding uninjured cell samples.

### 3.8. Immunoprecipitation

Jurkat cells were treated for 24 h and the conditioned media collected. Fifteen microlitres of Protein-G/-A beads (as a 50% slurry) was added to 1.3 ml of conditioned media and the mixture gently rotated (30 min, 4°C). The beads were centrifuged (2 min, 3000*g*) and discarded; 20 µl of Protein-G/-A beads + 1 µg of anti-MCM7 antibody was added to the supernatant and the mixture gently rotated (3 h, 4°C). The beads were centrifuged (2 min, 3000*g*) and kept as the ‘MCM7 pulldown’ material; 20 µl of Protein-G/-A beads + 1 µg of anti-FUS antibody was added to the supernatant and the mixture gently rotated (3 h, 4°C). The beads were centrifuged (2 min, 3000*g*) and kept as the ‘FUS pulldown’ material. The beads were boiled for 10 min in 35 µl of SDS-loading buffer with dithiothreitol and the supernatants subjected to SDS-PAGE/immunoblot analysis. Quantitation of the immunoprecipitation signal was performed using ImageQuant v. 5.2 software (Molecular Dynamics) and expressed relative to the total amount of FUS present in the Triton-soluble lysate from uninjured Jurkat cells.

### 3.9. Immunofluorescence

#### 3.9.1. Immunofluorescence for maleimide co-staining

Twenty-four hour-treated Jurkat cells were fixed in 2% paraformaldehyde with 40 mM chloroacetamide (4°C, 40 min, in dark). Cells were immobilized onto Superfrost slides (Tharmac Cellspin II, 320 g, 5 min) and incubated in 4% paraformaldehyde with 40 mM chloroacetamide (5 min, in dark). Cells were incubated in TBS-T (100 mM Tris–HCl pH 8.0 + 150 mM NaCl + 0.05% Tween20) with 40 mM chloroacetamide (20 min in dark). Cells were washed then incubated in TBS-T with 20 mM TCEP (20 min in dark). Cells were washed then incubated in TBS-T with 50 nM AlexaFluor488-conjugated maleimide (20 min in dark). Cells were washed then incubated in TBS-T with 5% FCS and 1 : 100 dilution of primary antibody (overnight, 4°C in dark). Cells were washed then incubated in TBS-T with 5% FCS and 1 : 1000 dilution of secondary antibody (AlexaFluor 568-conjugated donkey anti-mouse/goat IgG and CF647-conjugated donkey anti-rabbit IgG (Biotium), 2 h, in dark). Cells were stained in 10 mg l^−1^ Hoechst 33342, washed in TBS-T then mounted in Fluorescent Mounting Media (DAKO). Cells were imaged on a Nikon A1r-si resonant scanning confocal system (microscope: Nikon Ti, Tokyo, Japan; objective: Apo LWD, ×40 magnification, 1.15 numerical aperture, water immersion; sequential excitation: 405, 488, 561 and 638 nm lasers; emission: 450/50, 525/50, 595/50 and 700/75 nm filters; acquisition software: NIS Elements Advanced Research, Tokyo, Japan). Images were processed using ImageJ v. 1.50d software (National Institute of Health).

#### 3.9.2. Immunofluorescence for super-resolution microscopy

Jurkat cells were immunostained as above but without Hoechst 33 342 counterstaining. Cells were mounted in ProLong Diamond Antifade Mountant (Life Technologies) using high precision #1.5 glass coverslips (0.17 ± 0.01 mm; Menzel-Gläser). Samples were imaged by Stimulated Emission Depletion microscopy using a Leica TCS SP8 STED 3× platform (microscope: DMi8 inverted, objective: HC PL APO CS2 100×/1.40 oil, excitation: tunable pulsed white light laser, depletion: 775 nm and 592 nm depletion lasers, detection: sequential HyD detectors; software: LAS AF X). Images were processed using ImageJ v. 1.50d software (National Institute of Health).

### 3.10. Protein property analysis

#### 3.10.1. Protein sequence analysis

Canonical FASTA sequences for the different protein subsets (electronic supplementary material, table S1) were retrieved from UniProt and entered into the Sequence Manipulation Suite [46] to determine amino acid content, hydrophobicity and molecular weight.

#### 3.10.2. Protein aggregation prediction

Canonical FASTA sequences for the different protein subsets (electronic supplementary material, table S1) were retrieved from UniProt and uploaded to the AGGRESCAN [33] site to ascertain the predicted aggregation propensity. Note, only proteins with less than 1000 residues were analysed. AGGRESCAN provides the relative aggregation propensity of amino acids within both the central hydrophobic cluster of β-amyloid and within the context of a larger full-length soluble polypeptide [33]. AGGRESCAN's aggregation propensities are derived from *in vivo* experimental data [33]. Similar analyses (with similar results) were performed using the FoldAmyloid [39] and Zyggregator [40] databases (not shown).

#### 3.10.3. Intrinsic disorder content analysis

UniProt IDs (electronic supplementary material, table S1) were used to query the MobiDB 2.0 database [47]. The percentage of each protein in disorder (either from curated data or from consensus predicted data when no curated data existed) irrespective of segment was determined.

#### 3.10.4. Autoantigen analysis

UniProt IDs (electronic supplementary material, table S1) were cross-referenced against the full set of autoantigens from Backes *et al*. [36].

#### 3.10.5. Protein abundance analysis

The different protein subsets (electronic supplementary material, table S1) were uploaded to the PaxDB [48] site and their organismal abundance was retrieved from the Human PaxDB integrated dataset.

#### 3.10.6. Protein age analysis

Protein age was determined using the ProteinHistorian [49] website, whereby protein subsets were individually analysed via the swisspfam-PTHR7_youngest database and the Dollo parsimony reconstruction algorithm.

#### 3.10.7. Native disulfide bond analysis

Annotations from the UniProt [50] database were used to determine the number and location of native disulfide bonds for each protein of interest.

#### 3.10.8. Ontology enrichment analysis

Cytoscape v. 3.2.0 and the ClueGO v. 2.2.5 and CluePedia v. 1.2.5 plug-ins was used to perform ontology enrichment analysis as in [35]. Molecular function enrichment analysis was performed within GO term levels 3–5. Cellular compartment enrichment analysis was performed within GO term levels 4–10.

#### 3.10.9. Protein–protein interaction analysis

The high-confidence NCC-participating protein subset was entered into the Mentha [51] interactome database. All human interacting proteins were extracted and visualized as a network using Cytoscape. Duplicate interactions were removed and the network rendered directionless. The topology and characteristics of the resultant network was analysed using the Network Analyser plug-in [52].

### 3.11. Statistical analyses

All results are based on three independent experiments unless stipulated otherwise. Analyses were performed with GraphPad Prism 6 with *p* < 0.05 considered statistically significant. The analysis employed for each cohort is stated in the respective figure legend.

## Supplementary Material

RSOB-16-0098.R2 - Supplementary Figures and legends

## Supplementary Material

Supplementary Table S1

## Supplementary Material

Supplementary Table S2

## Supplementary Material

Supplementary Table S3

## Data accessibility

All mass spectrometry files used in the manuscript will be deposited in mzML format on the ProteomeXchange website (http://www.proteomexchange.org/).

## References

[1] Chiti F, Dobson CM 2009 Amyloid formation by globular proteins under native conditions. Nat. Chem. Biol. 5, 15–22. (doi:10.1038/nchembio.131) 19088715 10.1038/nchembio.131

[2] Mirabelli F, Salis A, Perotti M, Taddei F, Bellomo G, Orrenius S 1988 Alterations of surface morphology caused by the metabolism of menadione in mammalian cells are associated with the oxidation of critical sulfhydryl groups in cytoskeletal proteins. Biochem. Pharmacol. 37, 3423–3427. (doi:10.1016/0006-2952(88)90691-0) 3421993 10.1016/0006-2952(88)90691-0

[3] Hinshaw DB, Sklar LA, Bohl B, Schraufstatter IU, Hyslop PA, Rossi MW, Spragg RG, Cochrane CG 1986 Cytoskeletal and morphologic impact of cellular oxidant injury. Am. J. Pathol. 123, 454–464. 3717299 PMC1888261

[4] Hinshaw DB, Armstrong BC, Burger JM, Beals TF, Hyslop PA 1988 ATP and microfilaments in cellular oxidant injury. Am. J. Pathol. 132, 479–488. 3414780 PMC1880762

[5] Mirabelli F, Salis A, Marinoni V, Finardi G, Bellomo G, Thor H, Orrenius S 1988 Menadione-induced bleb formation in hepatocytes is associated with the oxidation of thiol groups in actin. Arch. Biochem. Biophys. 264, 261–269. (doi:10.1016/0003-9861(88)90593-0) 3395123 10.1016/0003-9861(88)90593-0

[6] Gabai VL, Kabakov AE, Mosin AF 1992 Association of blebbing with assembly of cytoskeletal proteins in ATP-depleted EL-4 ascites tumour cells. Tissue Cell 24, 171–177. (doi:10.1016/0040-8166(92)90090-T) 1589868 10.1016/0040-8166(92)90090-t

[7] Kabakov AE, Gabai VL 1993 Protein aggregation as primary and characteristic cell reaction to various stresses. Experientia 49, 706–710. (doi:10.1007/BF01923956) 8359277 10.1007/BF01923956

[8] Kabakov AE, Gabai VL 1994 Stress-induced insolubilization of certain proteins in ascites tumor cells. Arch. Biochem. Biophys. 309, 247–253. (doi:10.1006/abbi.1994.1109) 8135534 10.1006/abbi.1994.1109

[9] Kabakov AE, Budagova KR, Latchman DS, Kampinga HH 2002 Stressful preconditioning and HSP70 overexpression attenuate proteotoxicity of cellular ATP depletion. Am. J. Physiol. Cell Physiol. 283, C521–C534. (doi:10.1152/ajpcell.00503.2001) 12107062 10.1152/ajpcell.00503.2001

[10] Samson AL et al. 2012 Nucleocytoplasmic coagulation: an injury-induced aggregation event that disulfide crosslinks proteins and facilitates their removal by plasmin. Cell Rep. 2, 889–901. (doi:10.1016/j.celrep.2012.08.026) 23041318 10.1016/j.celrep.2012.08.026

[11] Nakajima H, Amano W, Fujita A, Fukuhara A, Azuma YT, Hata F, Inui T, Takeuchi T 2007 The active site cysteine of the proapoptotic protein glyceraldehyde-3-phosphate dehydrogenase is essential in oxidative stress-induced aggregation and cell death. J. Biol. Chem. 282, 26 562–26 574. (doi:10.1074/jbc.M704199200) 10.1074/jbc.M704199200 17613523

[12] Samson AL et al. 2014 Oxidation of an exposed methionine instigates the aggregation of glyceraldehyde-3-phosphate dehydrogenase. J. Biol. Chem. 289, 26 922–26 936. (doi:10.1074/jbc.M114.570275) 10.1074/jbc.M114.570275 PMC4175333 25086035

[13] Huang J, Hao L, Xiong N, Cao X, Liang Z, Sun S, Wang T 2009 Involvement of glyceraldehyde-3-phosphate dehydrogenase in rotenone-induced cell apoptosis: relevance to protein misfolding and aggregation. Brain Res. 1279, 1–8. (doi:10.1016/j.brainres.2009.05.011) 19445904 10.1016/j.brainres.2009.05.011

[14] Cumming RC, Schubert D 2005 Amyloid-beta induces disulfide bonding and aggregation of GAPDH in Alzheimer's disease. FASEB J. 19, 2060–2062. (doi:10.1096/fj.05-4195fje) 16186172 10.1096/fj.05-4195fje

[15] Nakajima H et al. 2009 Glyceraldehyde-3-phosphate dehydrogenase aggregate formation participates in oxidative stress-induced cell death. J. Biol. Chem. 284, 34 331–34 341. (doi:10.1074/jbc.M109.027698) 10.1074/jbc.M109.027698 PMC2797201 19837666

[16] Snider NT, Weerasinghe SV, Singla A, Leonard JM, Hanada S, Andrews PC, Lok AS, Bishr Omary M 2011 Energy determinants GAPDH and NDPK act as genetic modifiers for hepatocyte inclusion formation. J. Cell Biol. 195, 217–229. (doi:10.1083/jcb.201102142) 22006949 10.1083/jcb.201102142 PMC3198167

[17] Dedov VN, Dedova IV, Nicholson GA 2004 Hypoxia causes aggregation of serine palmitoyltransferase followed by non-apoptotic death of human lymphocytes. Cell Cycle 3, 1271–1277. (doi:10.4161/cc.3.10.1163) 15467453 10.4161/cc.3.10.1163

[18] Hu BR, Janelidze S, Ginsberg MD, Busto R, Perez-Pinzon M, Sick TJ, Siesjö BK, Liu CL 2001 Protein aggregation after focal brain ischemia and reperfusion. J. Cereb. Blood Flow Metab. 21, 865–875. (doi:10.1097/00004647-200107000-00012) 11435799 10.1097/00004647-200107000-00012

[19] Liu CL, Martone ME, Hu BR 2004 Protein ubiquitination in postsynaptic densities after transient cerebral ischemia. J. Cereb. Blood Flow Metab. 24, 1219–1225. (doi:10.1097/01.WCB.0000136706.77918.21) 15545915 10.1097/01.WCB.0000136706.77918.21 PMC3518068

[20] Samson AL, Borg RJ, Niego B, Wong CH, Crack PJ, Yongqing T, Medcalf RL 2009 A nonfibrin macromolecular cofactor for tPA-mediated plasmin generation following cellular injury. Blood 114, 1937–1946. (doi:10.1182/blood-2009-02-203448) 19584397 10.1182/blood-2009-02-203448

[21] Borg RJ et al. 2015 Dendritic cell-mediated phagocytosis but not immune activation is enhanced by plasmin. PLoS ONE 10, e0131216 (doi:10.1371/journal.pone.0131216) 26132730 10.1371/journal.pone.0131216 PMC4488505

[22] Kim CH et al. 2010 Role of reactive oxygen species-dependent protein aggregation in metabolic stress-induced necrosis. Int. J. Oncol. 37, 97–102. 20514401 10.3892/ijo_00000657

[23] Schaefer L 2014 Complexity of danger: the diverse nature of damage-associated molecular patterns. J. Biol. Chem. 289, 35 237–35 245. (doi:10.1074/jbc.R114.619304) 10.1074/jbc.R114.619304 PMC4271212 25391648

[24] t Hart BA, Chalan P, Koopman G, Boots AM 2013 Chronic autoimmune-mediated inflammation: a senescent immune response to injury. Drug Discov. Today 18, 372–379. (doi:10.1016/j.drudis.2012.11.010) 23195330 10.1016/j.drudis.2012.11.010

[25] Vogelgesang A, Dressel A 2011 Immunological consequences of ischemic stroke: immunosuppression and autoimmunity. J. Neuroimmunol. 231, 105–110. (doi:10.1016/j.jneuroim.2010.09.023) 20970200 10.1016/j.jneuroim.2010.09.023

[26] Al-Allaf AW, Sanders PA, Ogston SA, Marks JS 2001 A case-control study examining the role of physical trauma in the onset of rheumatoid arthritis. Rheumatology 40, 262–266. (doi:10.1093/rheumatology/40.3.262) 11285372 10.1093/rheumatology/40.3.262

[27] Pattison E, Harrison BJ, Griffiths CE, Silman AJ, Bruce IN 2008 Environmental risk factors for the development of psoriatic arthritis: results from a case-control study. Ann. Rheum. Dis. 67, 672–676. (doi:10.1136/ard.2007.073932) 17823200 10.1136/ard.2007.073932

[28] Gaipl US et al. 2007 Clearance deficiency and systemic lupus erythematosus (SLE). J. Autoimmun. 28, 114–121. (doi:10.1016/j.jaut.2007.02.005) 17368845 10.1016/j.jaut.2007.02.005

[29] de Scheerder IK, de Buyzere ML, Delanghe JR, Clement DL, Wieme RJ 1989 Anti-myosin humoral immune response following cardiac injury. Autoimmunity 4, 51–58. (doi:10.3109/08916938909034359) 2491642 10.3109/08916938909034359

[30] Nagata S, Hanayama R, Kawane K 2010 Autoimmunity and the clearance of dead cells. Cell 140, 619–630. (doi:10.1016/j.cell.2010.02.014) 20211132 10.1016/j.cell.2010.02.014

[31] Zhang G, Neubert TA 2009 Use of stable isotope labeling by amino acids in cell culture (SILAC) for phosphotyrosine protein identification and quantitation. Methods Mol. Biol. 527, 79–92. (doi:10.1007/978-1-60327-834-8_7) 10.1007/978-1-60327-834-8_7 PMC3757925 19241007

[32] Ong SE, Mann M 2006 A practical recipe for stable isotope labeling by amino acids in cell culture (SILAC). Nat. Protoc. 1, 2650–2660. (doi:10.1038/nprot.2006.427) 17406521 10.1038/nprot.2006.427

[33] Conchillo-Sole O, de Groot NS, Aviles FX, Vendrell J, Daura X, Ventura S 2007 AGGRESCAN: a server for the prediction and evaluation of ‘hot spots’ of aggregation in polypeptides. BMC Bioinformatics 8, 65 (doi:10.1186/1471-2105-8-65) 17324296 10.1186/1471-2105-8-65 PMC1828741

[34] Olzscha H et al. 2011 Amyloid-like aggregates sequester numerous metastable proteins with essential cellular functions. Cell 144, 67–78. (doi:10.1016/j.cell.2010.11.050) 21215370 10.1016/j.cell.2010.11.050

[35] Bindea G et al. 2009 ClueGO: a Cytoscape plug-in to decipher functionally grouped gene ontology and pathway annotation networks. Bioinformatics 25, 1091–1093. (doi:10.1093/bioinformatics/btp101) 19237447 10.1093/bioinformatics/btp101 PMC2666812

[36] Backes C, Ludwig N, Leidinger P, Harz C, Hoffmann J, Keller A, Meese E, Lenhof H-P 2011 Immunogenicity of autoantigens. BMC Genomics 12, 340 (doi:10.1186/1471-2164-12-340) 21726451 10.1186/1471-2164-12-340 PMC3149588

[37] Kwiatkowski TJ Jr et al. 2009 Mutations in the FUS/TLS gene on chromosome 16 cause familial amyotrophic lateral sclerosis. Science 323, 1205–1208. (doi:10.1126/science.1166066) 19251627 10.1126/science.1166066

[38] Kramer PA, Ravi S, Chacko B, Johnson MS, Darley-Usmar VM 2014 A review of the mitochondrial and glycolytic metabolism in human platelets and leukocytes: implications for their use as bioenergetic biomarkers. Redox Biol. 2, 206–210. (doi:10.1016/j.redox.2013.12.026) 24494194 10.1016/j.redox.2013.12.026 PMC3909784

[39] Garbuzynskiy SO, Lobanov MY, Galzitskaya OV 2010 FoldAmyloid: a method of prediction of amyloidogenic regions from protein sequence. Bioinformatics 26, 326–332. (doi:10.1093/bioinformatics/btp691) 20019059 10.1093/bioinformatics/btp691

[40] Pawar AP, Dubay KF, Zurdo J, Chiti F, Vendruscolo M, Dobson CM 2005 Prediction of ‘aggregation-prone’ and ‘aggregation-susceptible’ regions in proteins associated with neurodegenerative diseases. J. Mol. Biol. 350, 379–392. (doi:10.1016/j.jmb.2005.04.016) 15925383 10.1016/j.jmb.2005.04.016

[41] Havugimana PC et al. 2012 A census of human soluble protein complexes. Cell 150, 1068–1081. (doi:10.1016/j.cell.2012.08.011) 22939629 10.1016/j.cell.2012.08.011 PMC3477804

[42] Nielsen ML, Vermeulen M, Bonaldi T, Cox J, Moroder L, Mann M 2008 Iodoacetamide-induced artifact mimics ubiquitination in mass spectrometry. Nat. Methods 5, 459–460. (doi:10.1038/nmeth0608-459) 18511913 10.1038/nmeth0608-459

[43] Alonso H, Kleifeld O, Yeheskel A, Ong PC, Liu YC, Stok JE, De Voss JJ, Roujeinikova A 2014 Structural and mechanistic insight into alkane hydroxylation by *Pseudomonas putida* AlkB. Biochem. J. 460, 283–293. (doi:10.1042/BJ20131648) 24646189 10.1042/BJ20131648

[44] Au AE, Sashindranath M, Borg RJ, Kleifeld O, Andrews RK, Gardiner EE, Medcaff RL, Samson AL 2014 Activated platelets rescue apoptotic cells via paracrine activation of EGFR and DNA-dependent protein kinase. Cell Death Dis. 5, e1410 (doi:10.1038/cddis.2014.373) 25210793 10.1038/cddis.2014.373 PMC4540201

[45] Cox J, Mann M 2008 MaxQuant enables high peptide identification rates, individualized p.p.b.-range mass accuracies and proteome-wide protein quantification. Nat. Biotechnol. 26, 1367–1372. (doi:10.1038/nbt.1511) 19029910 10.1038/nbt.1511

[46] Stothard P 2000 The sequence manipulation suite: JavaScript programs for analyzing and formatting protein and DNA sequences. BioTechniques 28, 1102, 1104. 10868275 10.2144/00286ir01

[47] Di Domenico T, Walsh I, Martin AJ, Tosatto SC 2012 MobiDB: a comprehensive database of intrinsic protein disorder annotations. Bioinformatics 28, 2080–2081. (doi:10.1093/bioinformatics/bts327) 22661649 10.1093/bioinformatics/bts327

[48] Wang M, Weiss M, Simonovic M, Haertinger G, Schrimpf SP, Hengartner MO, von Mering C 2012 PaxDb, a database of protein abundance averages across all three domains of life. Mol. Cell. Proteomics 11, 492–500. (doi:10.1074/mcp.O111.014704) 22535208 10.1074/mcp.O111.014704 PMC3412977

[49] Capra JA, Williams AG, Pollard KS 2012 ProteinHistorian: tools for the comparative analysis of eukaryote protein origin. PLoS Comput. Biol. 8, e1002567 (doi:10.1371/journal.pcbi.1002567) 22761559 10.1371/journal.pcbi.1002567 PMC3386163

[50] UniProt C 2014 Activities at the universal protein resource (UniProt). Nucleic Acids Res. 42(Database issue), D191–D198. (doi:10.1093/nar/gkt1140) 24253303 10.1093/nar/gkt1140 PMC3965022

[51] Calderone A, Castagnoli L, Cesareni G 2013 Mentha: a resource for browsing integrated protein-interaction networks. Nat. Methods 10, 690–691. (doi:10.1038/nmeth.2561) 23900247 10.1038/nmeth.2561

[52] Assenov Y, Ramirez F, Schelhorn SE, Lengauer T, Albrecht M 2008 Computing topological parameters of biological networks. Bioinformatics 24, 282–284. (doi:10.1093/bioinformatics/btm554) 18006545 10.1093/bioinformatics/btm554

