# Supplementary material for: Physicochemical properties that control protein aggregation also determine whether a protein is retained or released from necrotic cells
Source: Open Biol. 2016 Nov 3;6(11):160098. doi: 10.1098/rsob.160098 (PMC5133435; doi:10.1098/rsob.160098)
Supplement: RSOB-16-0098.R2 - Supplementary Figures and legends [file rsob160098supp1.docx]

Supplementary material for “Physicochemical properties that control protein aggregation also determine whether a protein is retained or released from necrotic cells” by Samson et al., (2016) *Open Biol.* **6:** 160098.

**Supplementary Figures & Legends:**


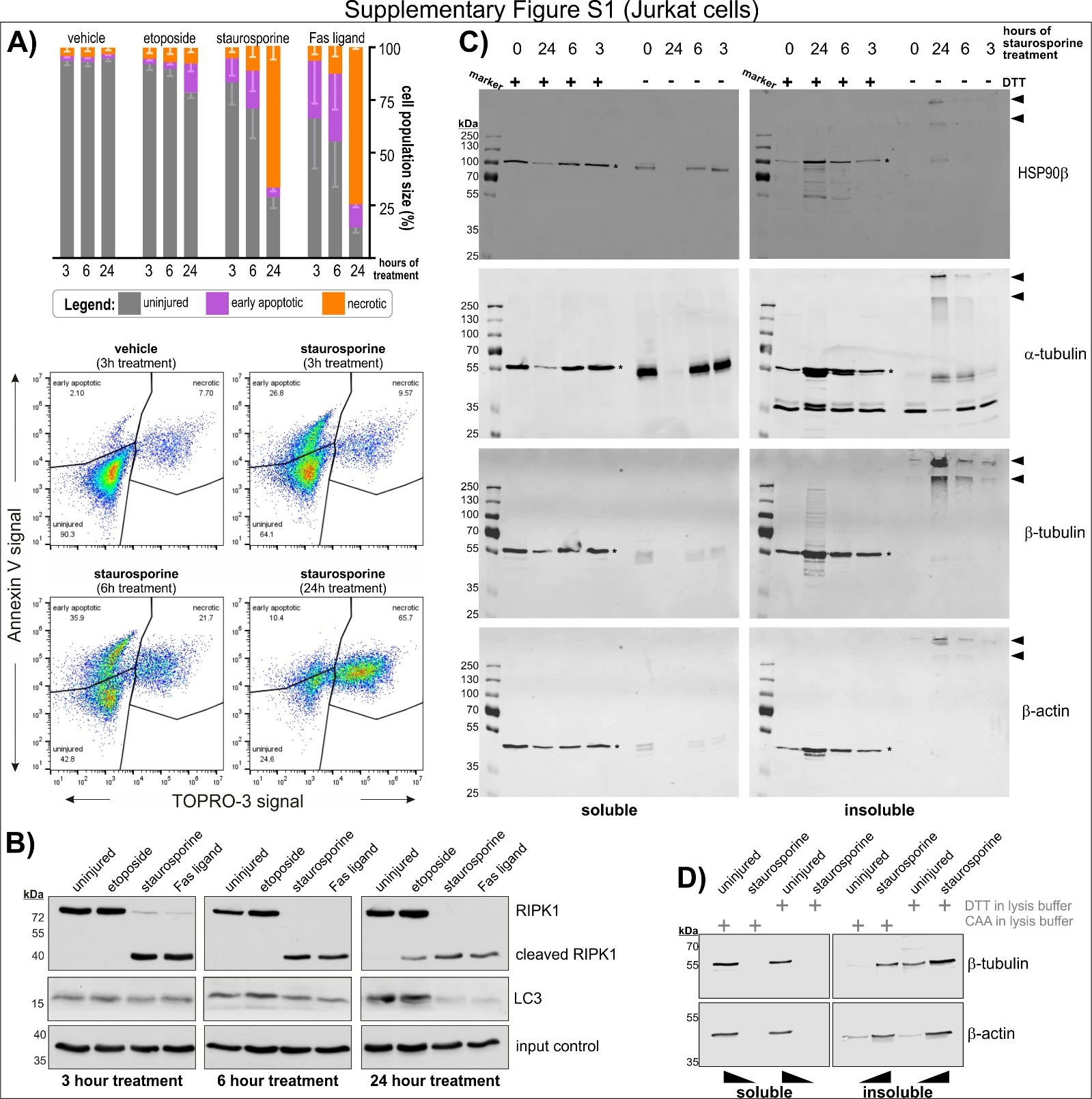


**Supplementary Figure S1 - Secondary necrosis of human Jurkat lymphocytes causes NCC.**

**A)** Jurkat cells were treated with vehicle, etoposide, staurosporine or Fas ligand. At increasing time points, the proportion of uninjured (AnnexinV^AlexaFluor488^-negative TO-PRO-3-negative; grey bars), early apoptotic (AnnexinV ^AlexaFluor488^-positive TO-PRO-3-negative; purple bars) and secondary necrotic cells (AnnexinV ^AlexaFluor488^-positive TO-PRO-3-positive; orange bars) was determined by flow cytometry. The bar graph shows the proportion of each subpopulation collated across multiple independent experiments (n=3-7; mean with sem). Also shown is raw scatter plotted data of a single representative timecourse of staurosporine-treated Jurkat cells with the relevant subpopulation gates superimposed. **B)** Jurkat cells were treated with vehicle (uninjured), etoposide, staurosporine or Fas ligand. At increasing time points, whole cell protein lysates were prepared and subjected to SDS-PAGE under reducing conditions followed by immunoblot analysis for Receptor-interacting serine/threonine-protein kinase 1 (RIPK1), LC3 and GAPDH (as an input control). Full-length RIPK1 is needed for regulated necrosis, whereas caspase-mediated RIPK1 cleavage occurs during apoptosis (1). LC3 cleavage is characteristic of autophagic cell death. **C)** Jurkat cells were treated with vehicle (uninjured) or staurosporine. At increasing time points, Triton-soluble and -insoluble proteins were isolated and subjected to SDS-PAGE in the presence/absence of dithiothreitol (DTT) followed by immunoblot analysis. Asterisks demarcate monomers and arrowheads demarcate disulfide-crosslinked aggregates of the NCC-participating proteins. **D)** Jurkat cells were treated with vehicle (uninjured) or staurosporine for 24 hours then whole cell protein lysates in PBS + 1% Tx100 supplemented with either 50mM chloroacetamide (CAA) or 100mM diothiothreitol (DTT). Triton-soluble and -insoluble proteins were isolated and subjected to SDS-PAGE in the presence of DTT followed by immunoblot analysis. Black triangles indicate that β-tubulin and β-actin (two established NCC-proteins) both transition from a soluble form into an insoluble form upon injury.


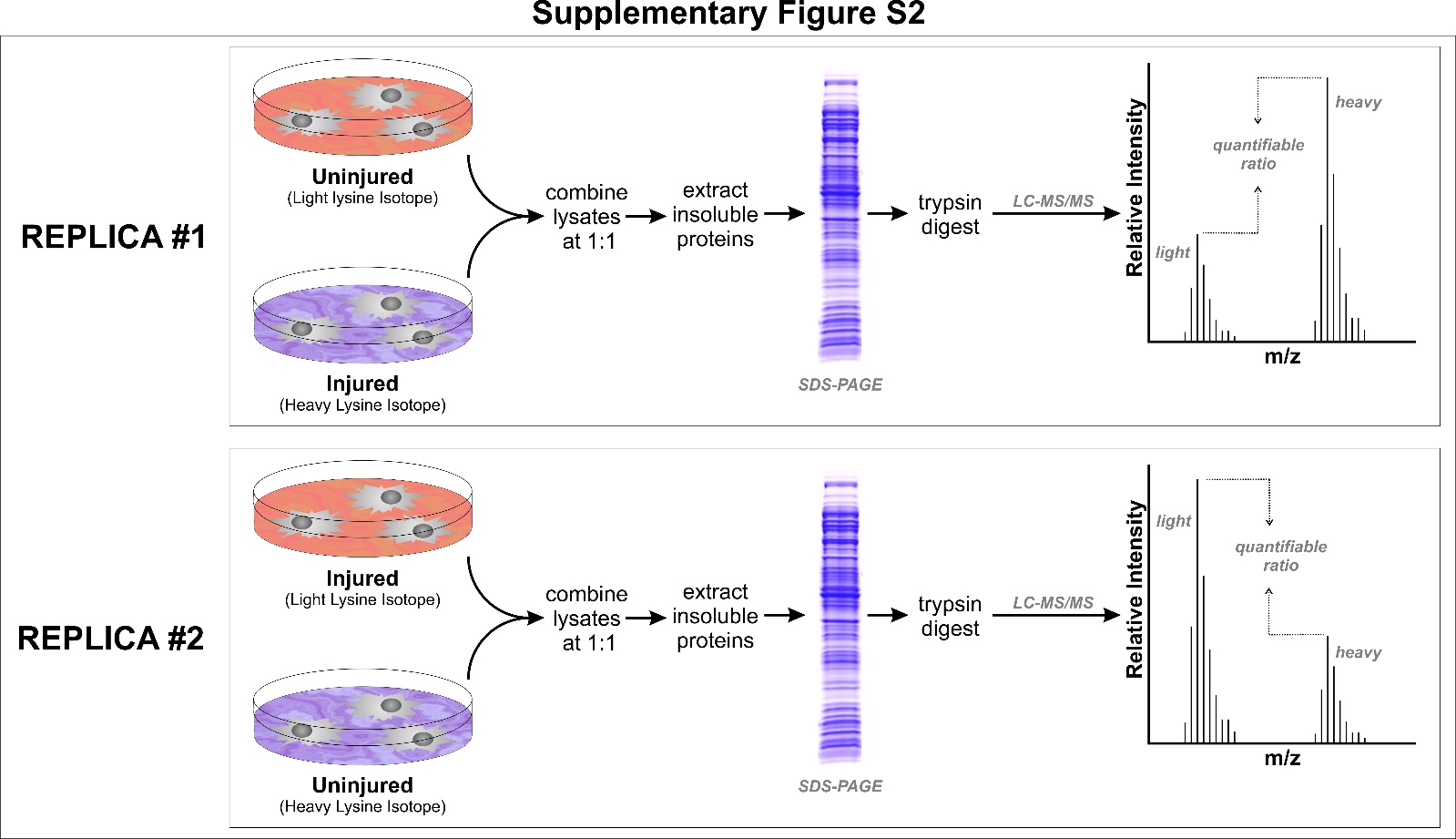


**Supplementary Figure S2 – Cartoon of SILAC experiment design.**

Two replicas were performed per SILAC experiment. In one replica, ‘light’ lysine was incorporated into an uninjured (reference) culture and ‘heavy’ lysine was incorporated into a separate culture prior to injury. In the second replica, ‘heavy’ lysine was incorporated into an uninjured (another reference) culture and ‘light’ lysine was incorporated into a separate culture prior to injury. Whole cell lysates were harvested from all cultures and each lysate was combined at a 1:1 ratio with a reference cell lysate harbouring the opposing lysine isotope. The insoluble material from the combined lysate was then extracted, fractionated by SDS-PAGE, digested and analysed via mass spectrometry. Quantitation of relative peptide abundance was based on the ratio of peak intensities between ‘light’ and ‘heavy’ versions of the same peptide. Note, only proteins quantified in both replicas were considered.


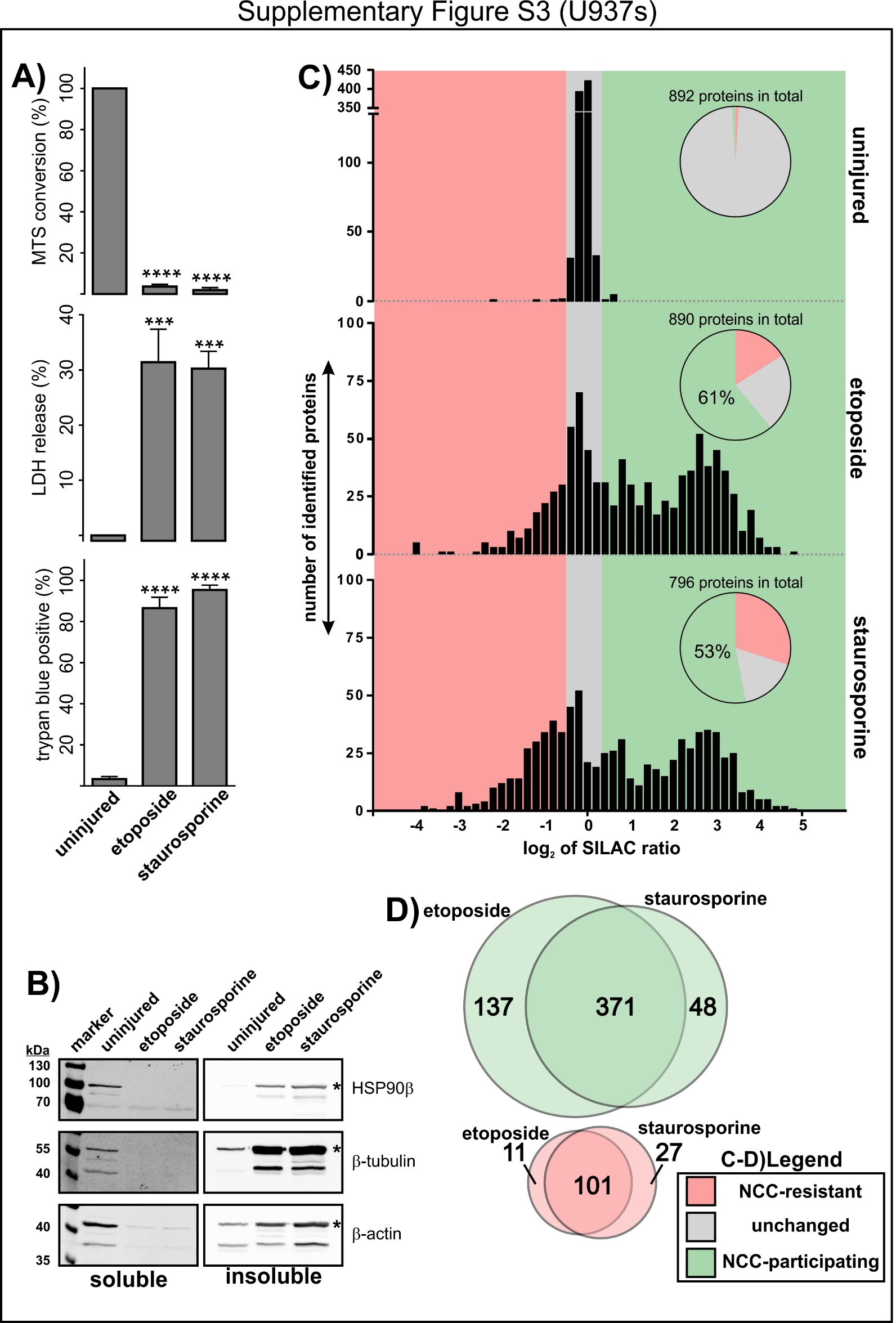


**Supplementary Figure S3 -** **Similar changes in the abundance of insoluble intracellular proteins in necrotic human U937 monocytes.**

**A-B)** Human U937 monocytes were treated with vehicle (uninjured) or injured with etoposide or staurosporine for 24 hours. **A)** Metabolism (% MTS; top graph) and necrosis (% LDH and % trypan blue positive; middle and bottom graphs respectively) were then measured. Data are presented as mean + sem from n=3 independent experiments. ***p<0.001 and ****p<0.0001 relative to uninjured cultures as determined by one-way ANOVA with Newman-Keuls correction. **B)** Triton-soluble and -insoluble proteins were isolated and subjected to SDS-PAGE under reducing conditions followed by immunoblot analysis. Asterisks demarcate monomers of the NCC-participating proteins. Results are representative of n=3 independent experiments. **C)** U937 cultures labelled with either heavy or light lysine isotopes were treated with vehicle (uninjured), etoposide or staurosporine for 24 hours. Triton-insoluble fractions were extracted and proteins identified using quantitative mass spectrometry. The abundance of identified proteins relative to that in isotopically-different uninjured cultures was then plotted as the log_2_ ratio of the stable isotope label (SILAC); where an increased ratio indicates more of a specific insoluble protein in injured cells relative to uninjured cells and a decreased ratio indicates less of a specific insoluble protein in injured cells relative to uninjured cells. Proteins with a log_2_ ratio >+0.6 were gated into the NCC-participating subproteome (green colour) and proteins with a log_2_ ratio <-0.6 were gated into the NCC-resistant subproteome (pink colour). Inserts show the percentage of identified proteins that fall within each gate. **D)** Partial overlap in the gated subproteomes between etoposide- and staurosporine-induced necrotic U937 cells. The number of proteins within each gate is shown.


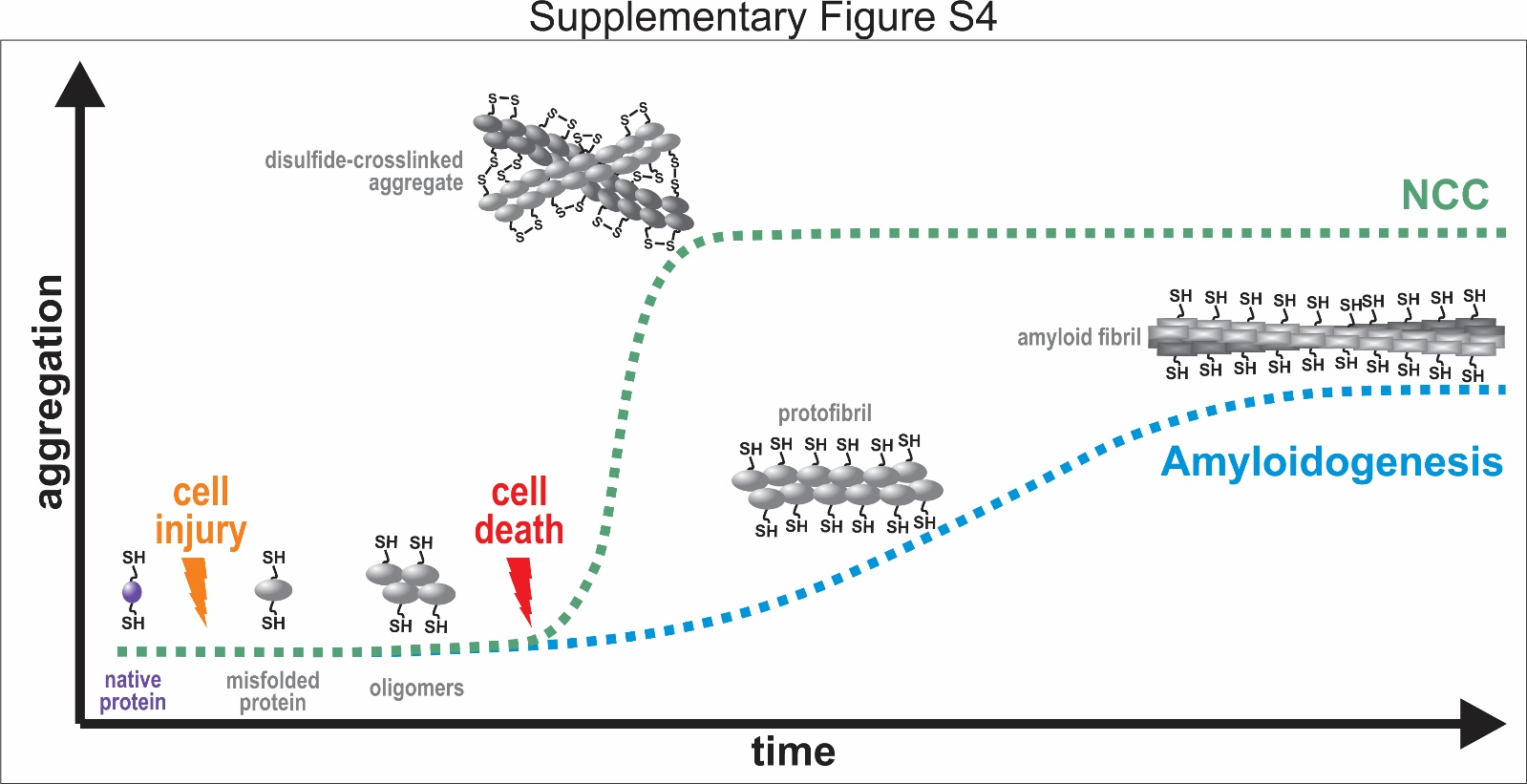


**Supplementary Figure S4 – NCC and amyloidogenesis are similar but distinct processes.**

Shown is a comparison of the “primary nucleation” model of amyloidogenesis (2) with our putative model of NCC. Amyloidogenesis features an initial lag phase (with misfolding then formation of low *n-*number oligomers) and a subsequent elongation phase (with formation of higher *n-*number oligomers, protofibrils and finally amyloid fibrils). As many of the primary sequence traits which drive amyloidogenesis are also present in NCC-participating proteins (Figure 4a), both processes likely follow similar principles during their initial stages. However, amyloid formation often occurs in the absence of (or independently of) disulfide bonding (3-5), whereas delayed disulfide-crosslinking of cysteine-rich proteins is a defining characteristic of NCC-aggregation (Figure S1c and (6)). Thus, we propose that en masse oxidation during late-stage cell death diverts oligomers away from the amyloid pathway and instead produces non-fibrillar high molecular weight disulfide-crosslinked aggregates. The rapid onset and end products of NCC are likely to be incongruent with amyloid; which instead relies upon non-covalent orderly association between discrete protein “hot spots” (7). These hypotheses are supported by our prior published data showing that NCC-aggregates exhibit a prefibrillar oligomeric conformation with no subsequent appearance of fibrillar species (6).


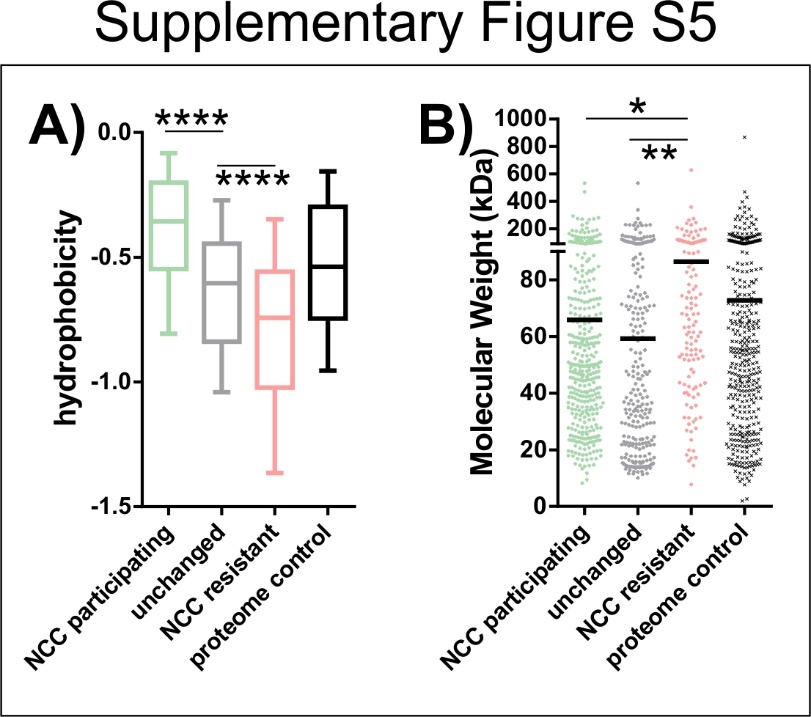


**Supplementary Figure S5 – NCC-participating proteins have distinct hydrophobicity and molecular weight profile.**

**A-B)** The same high confidence protein subsets and the randomly selected control subset from Figure 4 were further analysed. Graphs comparing the hydropathy **(A)** and the molecular weight **(B)** across the different protein subsets. *p<0.05, **p<0.01 and ****p<0.0001 as determined by one-way ANOVA with Tukey’s correction.


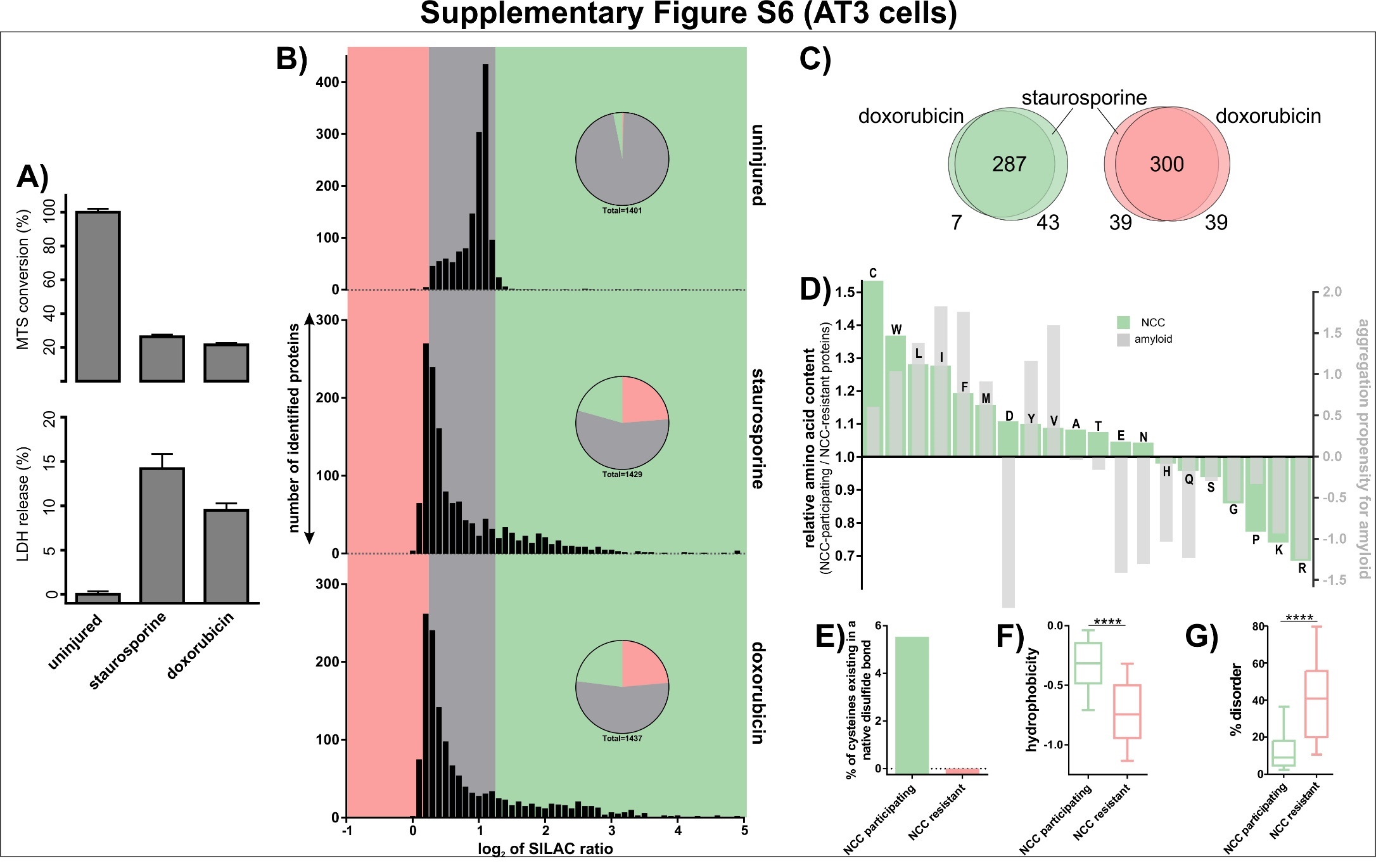


**Supplementary Figure S6 – Similar changes in the abundance of insoluble intracellular proteins in necrotic mouse AT3 cells.**

**A-B)** Mouse AT3 cells were treated with vehicle (uninjured) or injured with staurosporine or doxorubicin for 24 hours. **A)** Metabolism (% MTS; top graph) and necrosis (% LDH; bottom graph) were then measured. Data are presented as mean + sem from n=2 independent experiments. **B)** AT3 cultures labelled with either heavy or light lysine isotopes were treated with vehicle (uninjured), staurosporine or doxorubicin for 24 hours. Triton-insoluble fractions were extracted and proteins identified using quantitative mass spectrometry. The abundance of identified proteins relative to that in isotopically-different uninjured cultures was then plotted as the log_2_ ratio of the stable isotope label (SILAC); where an increased ratio indicates more of a specific insoluble protein in injured cells relative to uninjured cells and a decreased ratio indicates less of a specific insoluble protein in injured cells relative to uninjured cells. Proteins with a log_2_ ratio >+1.3 were gated into the NCC-participating subproteome (green colour) and proteins with a log_2_ ratio <+0.3 were gated into the NCC-resistant subproteome (pink colour). Inserts show the percentage of identified proteins that fall within each gate. Please note, a different gating strategy to that for Figure 1B and Figure S3C was used because the seeding of heavy and light AT3 populations was not 1:1 (a technical issue related to the strong “clumping” nature of AT3 cells). **C)** Partial overlap in the gated subproteomes between doxorubicin- and staurosporine-induced necrotic AT3 cells. **D)** Shown in green is the amino acid composition of NCC-participating proteins, relative to NCC-resistant proteins (arbitrarily normalised to 1) for necrotic AT3 cells. Shown in grey is the predicted relative contribution of individual amino acids to amyloidogenic aggregation as defined in AGGRESCAN(7) (arbitrarily normalised to 0). **E-G)** Graphs comparing the degree of native disulfide bonding **(E)**, the hydrophobicity **(F),** and the intrinsic disorder content **(G)** across the four protein subsets from necrotic AT3 cells.


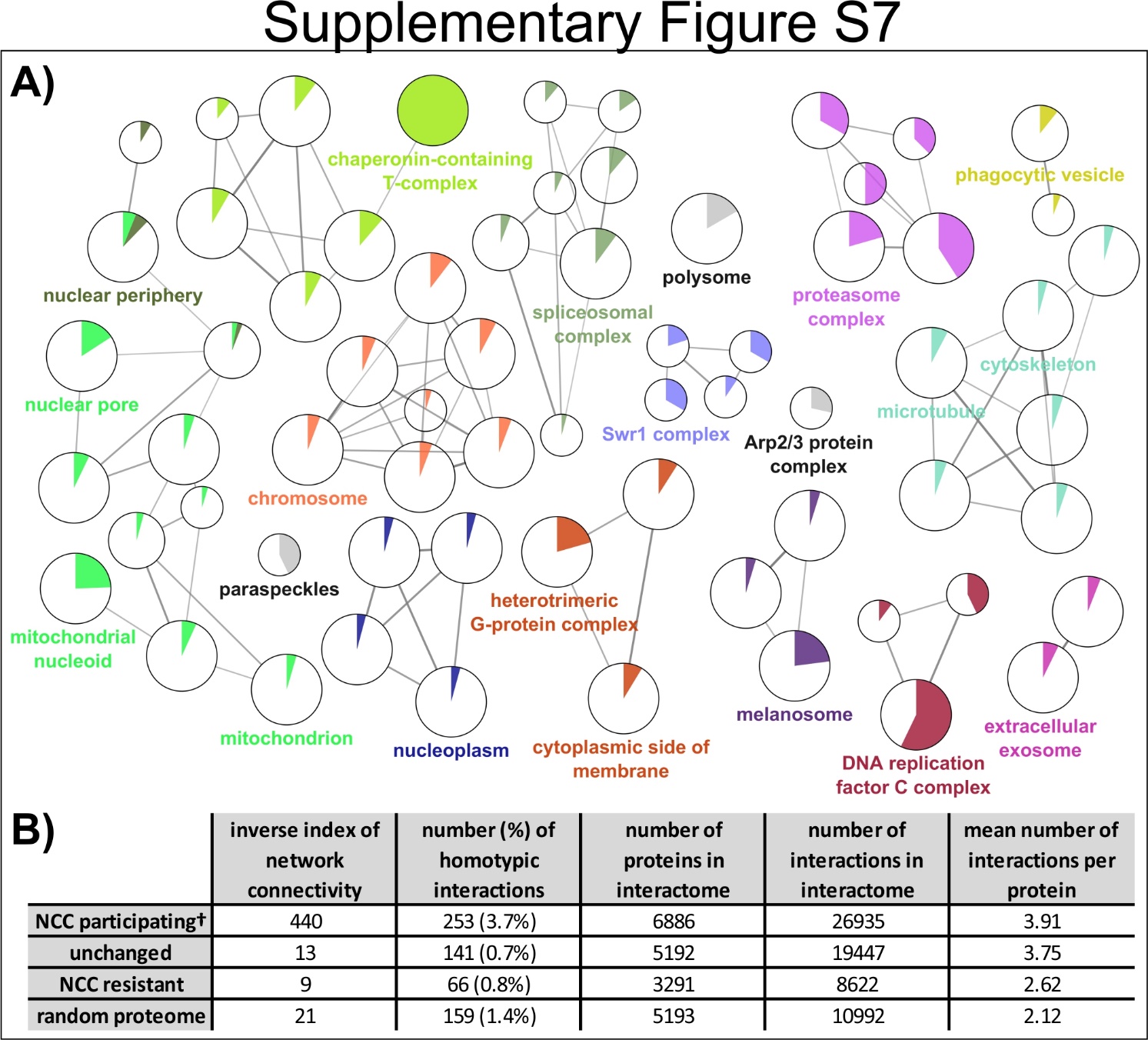


**Supplementary Figure S7 – higher order structural features of NCC.**

**A-B)** The same high confidence protein subsets and the randomly selected control subset from Figure 4 were further analysed. **A)** Network depicting the cellular compartments that were significantly overrepresented in the NCC-participating subset, relative to the complete human proteome. Nodes (circles) represent different cellular compartment ontologies. Related compartments are connected by edges (straight lines) and have been grouped using ClueGo(8) with a kappa score level of ≥0.4. The node size is proportional to the statistical significance of enrichment. The coloured pie segments which fill each node indicate the percent of the ontology that appears in the NCC-participating subset. The complete list of enriched cellular compartments (with statistical significance values) for both the NCC-participating and NCC-resistant subsets is provided in Table S3. **B)** Human proteins which interacted with NCC-participating proteins were extracted from the Mentha(9) database and the resultant interactome subjected to network analysis(10). The table shows the most pertinent results from this network analysis. Note†: polyubiquitin was removed from the NCC-participating protein subset as it dramatically skewed the network topology.

**Supplementary References:**

1. Vandenabeele P, Declercq W, Van Herreweghe F, Vanden Berghe T. The role of the kinases RIP1 and RIP3 in TNF-induced necrosis. Science signaling. 2010;3(115):re4.

2. Knowles TP, Vendruscolo M, Dobson CM. The amyloid state and its association with protein misfolding diseases. Nat Rev Mol Cell Biol. 2014;15(6):384-96.

3. Li Y, Gong H, Sun Y, Yan J, Cheng B, Zhang X, et al. Dissecting the role of disulfide bonds on the amyloid formation of insulin. Biochem Biophys Res Commun. 2012;423(2):373-8.

4. Tanaka N, Morimoto Y, Noguchi Y, Tada T, Waku T, Kunugi S, et al. The mechanism of fibril formation of a non-inhibitory serpin ovalbumin revealed by the identification of amyloidogenic core regions. The Journal of biological chemistry. 2011;286(7):5884-94.

5. Ellisdon AM, Thomas B, Bottomley SP. The two-stage pathway of ataxin-3 fibrillogenesis involves a polyglutamine-independent step. The Journal of biological chemistry. 2006;281(25):16888-96.

6. Samson AL, Knaupp AS, Sashindranath M, Borg RJ, Au AE, Cops EJ, et al. Nucleocytoplasmic coagulation: an injury-induced aggregation event that disulfide crosslinks proteins and facilitates their removal by plasmin. Cell Rep. 2012;2(4):889-901.

7. Conchillo-Sole O, de Groot NS, Aviles FX, Vendrell J, Daura X, Ventura S. AGGRESCAN: a server for the prediction and evaluation of "hot spots" of aggregation in polypeptides. BMC bioinformatics. 2007;8:65.

8. Bindea G, Mlecnik B, Hackl H, Charoentong P, Tosolini M, Kirilovsky A, et al. ClueGO: a Cytoscape plug-in to decipher functionally grouped gene ontology and pathway annotation networks. Bioinformatics. 2009;25(8):1091-3.

9. Calderone A, Castagnoli L, Cesareni G. mentha: a resource for browsing integrated protein-interaction networks. Nature methods. 2013;10(8):690-1.

10. Assenov Y, Ramirez F, Schelhorn SE, Lengauer T, Albrecht M. Computing topological parameters of biological networks. Bioinformatics. 2008;24(2):282-4.
